# Efficient multi-task learning with adaptive temporal structure for progression prediction

**DOI:** 10.1007/s00521-023-08461-9

**Published:** 2023-05-10

**Authors:** Menghui Zhou, Yu Zhang, Tong Liu, Yun Yang, Po Yang

**Affiliations:** 1 Department of Software, Yunnan University, Kunming, 674199 Yunnan Province China; 2 Department of Computer Science, University of Sheffield, Sheffield, S10 2TT UK

**Keywords:** Multi-task learning, Progression prediction, Adaptive temporal structure

## Abstract

In this paper, we propose a novel efficient multi-task learning formulation for the class of progression problems in which its state will continuously change over time. To use the shared knowledge information between multiple tasks to improve performance, existing multi-task learning methods mainly focus on feature selection or optimizing the task relation structure. The feature selection methods usually fail to explore the complex relationship between tasks and thus have limited performance. The methods centring on optimizing the relation structure of tasks are not capable of selecting meaningful features and have a bi-convex objective function which results in high computation complexity of the associated optimization algorithm. Unlike these multi-task learning methods, motivated by a simple and direct idea that the state of a system at the current time point should be related to all previous time points, we first propose a novel relation structure, termed adaptive global temporal relation structure (AGTS). Then we integrate the widely used sparse group Lasso, fused Lasso with AGTS to propose a novel convex multi-task learning formulation that not only performs feature selection but also adaptively captures the global temporal task relatedness. Since the existence of three non-smooth penalties, the objective function is challenging to solve. We first design an optimization algorithm based on the alternating direction method of multipliers (ADMM). Considering that the worst-case convergence rate of ADMM is only sub-linear, we then devise an efficient algorithm based on the accelerated gradient method which has the optimal convergence rate among first-order methods. We show the proximal operator of several non-smooth penalties can be solved efficiently due to the special structure of our formulation. Experimental results on four real-world datasets demonstrate that our approach not only outperforms multiple baseline MTL methods in terms of effectiveness but also has high efficiency.

## Introduction

As a promising field, multi-task learning (MTL) [6] is a topic of interest to data mining, machine learning, natural language processing, and computer vision communities. Typically, MTL refers to learning multiple related prediction tasks simultaneously, rather than learning each task independently. Simultaneous learning enables the model to share common information among related tasks and acts as an inductive bias to improve generalization performance. It has led to many successful practical applications, such as entity recommendation [19], travel time estimation [24], image captioning [49], human action recognition [25], etc. One interesting example is harnessing MTL for predicting the number of infections and identifying key factors in the social measure for the COVID-19 pandemic. Considering the prediction of daily COVID-19 infections at a certain week as a single task, multiple tasks at different time points are intrinsically related, such that a joint analysis of multiple time points via multi-task learning is expected to improve the long-term prediction of the multiple-wave dynamic of the COVID-19 pandemic.

**Fig. 1:**
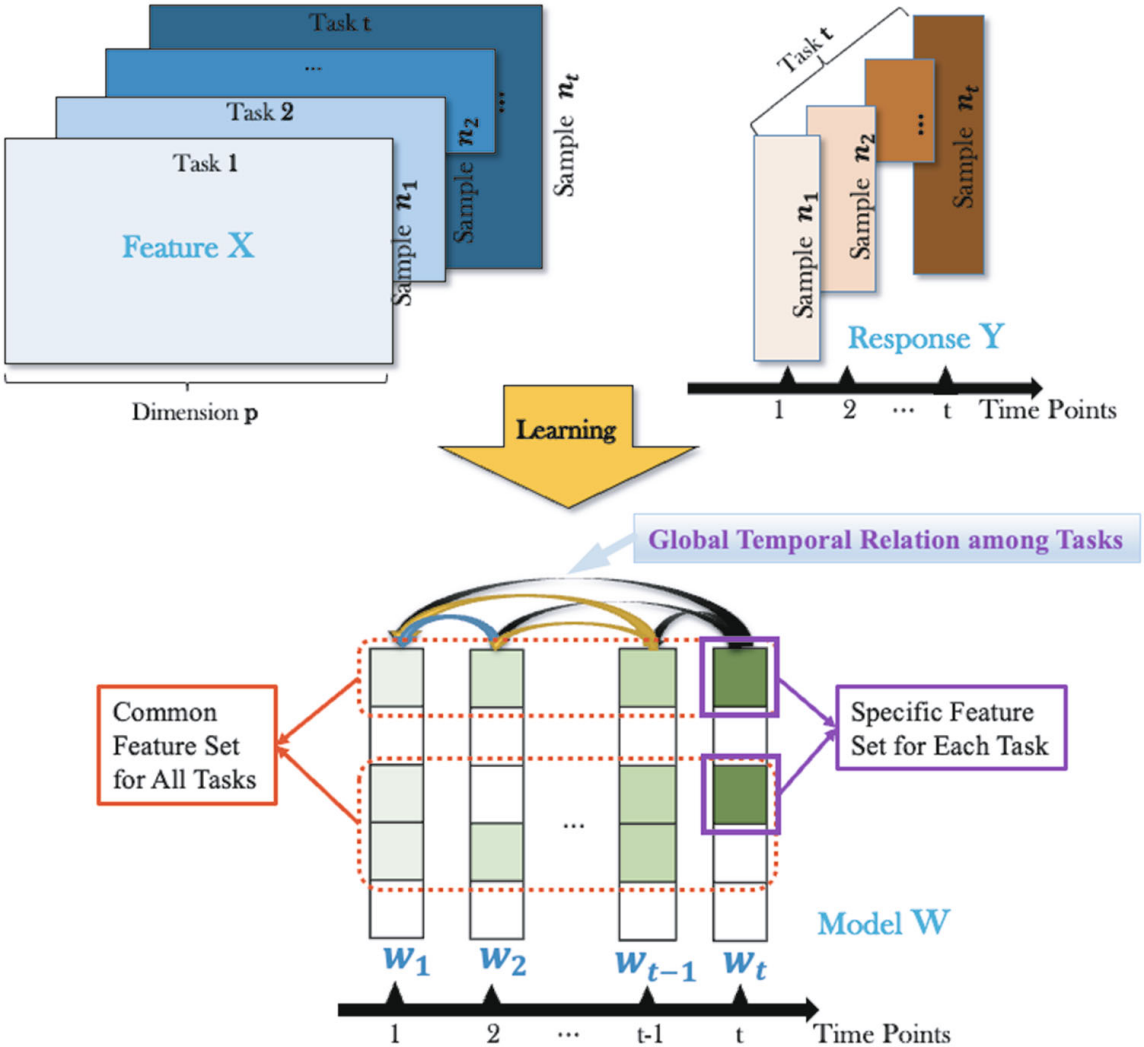
Illustration of MTL for progression problem. Assume we have a total of *t* time points, and each time point of a sequence of time points concerns a prediction task. Different task has corresponding different samples $X_i, i \in \{1,\cdots , t\}$, but with same feature set. Every time point is temporally related to its all previous time points, i.e., every task is related to all its previous tasks

However, in MTL research, it is challenging to know how the tasks are related and use concrete ways to capture the complex correlation among tasks [45]. Previous studies achieve these goals by employing effective feature selection approaches [41] or optimizing the relation structure of tasks [2, 23]. As for multi-task feature selection approaches, they are limited by a strict assumption that without considering differences between tasks, the selected features are shared among all tasks. Recent studies have suggested a more flexible approach that involves feature selection by decomposing a coefficient into a shared part and an individual part [21] or factorizing a coefficient using a feature-specific part and a task-specific part [41]. Nevertheless, this approach has limited ability to share common information due to the lacking use of complicated task relatedness.

Differing from feature selection ones, the task relation approaches, mainly consisting of low-rank assumption and task grouping structure, usually have unavoidable heavy computational costs. The low-rank approaches assume the coefficient vectors lie within a low-dimensional latent space, achieved by imposing a trace constraint [1] or encouraging sparsity on the singular values of the coefficient matrix [14]. However, this assumption cannot fully capture the complicated task correlation and the singular value decomposition requires heavy computational complexity. Some other task grouping methods decompose the model matrix into the product of two matrices [2] to capture task grouping structure but fail to perform feature selection. This decomposition way also leads to a bi-convex objective formulation that cannot guarantee to achieve the global minimum and needs to employ alternating optimization. It makes the associated algorithm has expensive computational complexity. Therefore, the main challenge is how to propose a novel multi-task learning method to not only perform feature selection but also capture the complex relationships among tasks, on the premise of ensuring high efficiency.

In this paper, we explore multi-task learning as an efficient solution for solving a series of progression problems in which the state will continuously change over time.

A starting point for our method is a direct and clear assumption that in the progression problem, the state at the current time point should be related to all previous time points, which can be considered as a kind of global temporal relatedness. Specifically, refer to Fig. 1, the prediction at each time point is treated as a task, and the coefficient matrix $W = [\varvec{w_1}, \cdots , \varvec{w_t}]$, $\varvec{w_k}$ is related to all previous tasks $\varvec{w_i}, i = [1, \cdots , k-1]$. We propose a novel ***A****daptive* ***G****lobal* ***T****emporal* ***S****tructure* (**AGTS**) to model this idea such that the relatedness matrix among tasks can be determined adaptively and the global temporal information is incorporated into our approach.

To enable the capability to perform feature selection, we prefer the widely used sparse group Lasso [36] which conduct simultaneous joint feature selection for all tasks and the selection of a specific feature set for each task. However, this penalty does not consider the relation among tasks. So we combine AGTS with the sparse group Lasso to propose a novel temporal sparse group Lasso. It not only performs feature selection, but also utilizes the global temporal relatedness among tasks. To further improve the capability of our method, we then combine the fused Lasso [38] with AGTS to propose a ***global temporal smoothness penalty*** which means the state of the progression problem will not fluctuate dramatically over time. Recently, the fused Lasso is extended from single-task learning to multi-task learning to chase the local temporal smoothness in [52, 54], which means the difference of the predictions between successive time points is small. However, this assumption only considers two adjacent time points, potentially missing out on helpful task dependencies beyond the immediate neighbours. In contrast, our global temporal smoothness penalty considers the adjacent time point as well as all previous time points.

By integrating the proposed temporal sparse group Lasso penalty and global temporal smoothness penalty, we present a novel convex multi-task learning formulation which takes into account the complex temporal relation among tasks while selecting important features. It is worth noting that compared to the bi-convex methods that concentrate on the task relation, our method utilizes a relation matrix to adaptively capture the temporal relatedness among all tasks, resulting in a convex objective function. This convexity is the key to designing an efficient optimization algorithm.

The proposed formulation is challenging to solve due to the utilization of three non-smooth penalties. We design the optimization algorithm based on the well-developed alternating direction method of multipliers (ADMM) [5]. Although ADMM is widely used in multi-task learning literature [22], the worst-case convergence rate of ADMM is only ${\mathcal {O}}({1}/{\sqrt{k}})$ for *k* iterations and the actual speed of the implementation may rely on the choice of the penalty parameter $\rho$ [43]. We then devise an efficient optimization algorithm based on the accelerated gradient method (AGM) [32] which has the optimal convergence rate for the class of first-order methods. The key step in using AGM is the computation of the proximal operator associated with the composite of non-smooth penalties, which is usually the most time-consuming block in the optimization algorithm. However, since the task relation matrix of AGTS is invertible, we can efficiently compute the proximal operator of our model depending on the decomposition property of the combination of fused Lasso and sparse group Lasso proved in [52].

The main contribution of this paper is concluded as follows:

- We propose a main assumption that for the class of progression problems as a sequence of time points, the state at the current time point is related to all previous time points. Based on it, we propose a novel adaptive global temporal structure, which adaptively captures the complex relatedness among multiple time points.
- One novel multi-task learning approach incorporating both effective feature selection or optimised task relation structure is formulated, with benefits to balance the trade-off of efficiency and effectiveness towards general MTL applications.
- A new ADMM-based algorithm is designed to solve our proposed MTL formulation. For tackling the worst-case convergence rate of ADMM, we exploit the special decomposition property of our formulation to propose the AGM-based algorithm with improved efficiency.
- Comprehensive experimental results on four real-world datasets demonstrate our approach not only outperforms multiple baseline MTL methods in terms of effectiveness, but also has high efficiency.

**Organization:** In Sect.  2, we discuss the related work. In Sect.  3, we present the proposed method of efficient multi-task learning with adaptive temporal structure. Our two optimization algorithms are detailed in Sect. 4. In Sect. 5, we report the empirical results, and we conclude this paper in Sect. 6.

## Related work

In this section, we briefly discuss the related MTL works on feature selection, task relation structure, and temporal multi-task learning.

### Feature selection methods

The feature selection approach is usually applied to select a subset of features for related tasks. It can be conducted by many kinds of sparsity-introducing penalties, e.g, Lasso, group Lasso $l_{1,2}$-norm, $l_{1,\infty }$-norm [26], sparse group Lasso [36] or other penalties with singularity property like Log-Exp-Sum penalty [11].

To further improve the model performance, some methods decompose the model coefficient matrix $W = P+U$ [12, 21]. Then various penalties are applied on the different parts to select features, e.g, [12] uses group Lasso to penalize *P* to select the features at group level while identifying the outlier tasks by penalizing the $L2-norm$ of every column of *U*.

### Task relation methods

The low-rank and task-grouping approaches both focus on complex task relation. Specially, the low-rank approach assumes the coefficient vectors lie in a low-dimensional latent space by imposing a trace constraint [1] or penalizing the singular value of the coefficient matrix [14, 30] with heavy complexity. But these methods might be too strict in practice since many task relation structures do not have low-rank property [31]. Compared to the low-rank method, some task grouping methods decompose the coefficient matrix $W = PU$ [23], leading to a bi-convex objective function which is challenging to achieve the global minimum and design an efficient optimization algorithm. [22] attempts to combine feature selection ability with task grouping structure, however, still has a bi-convex objective function and heavy computational complexity.

### Temporal multi-task learning

Some works use multi-task learning methods to predict Alzheimer’s disease progression [51, 52]. The key challenge is how to capture the temporal relation among tasks. [51] propose a temporal group Lasso formulation TGL which penalizes deviations between two adjacent tasks to chase temporal smoothness relation at the task level. [52] propose a fused sparse group Lasso formulation cFSGL in which a fusion penalty is used to penalize the difference of the feature weight at two successive time points to chase temporal smoothness at the feature level. However, both TGL and cFSGL only chase the local temporal smoothness, since they only consider the relation between neighbouring time points.

We conclude that our proposed approach has several main advantages:

- Compared to the feature selection methods, our approach not only conducts simultaneous joint feature selection for all tasks and selection of a specific feature set for each task, but also adaptively captures the intrinsic temporal task relation.
- Compared to the task relation methods with bi-convex objective function, our convex formulation can achieve the global minimum easily. The convexity of our formulation also enables us to solve the proximal operator of several penalties efficiently, which is the key step in designing an efficient AGM-based optimization algorithm.
- Compared to temporal multi-task learning, which only considers the local temporal relation, our approach chases the global temporal relation in an adaptive way.

## Methods

Consider we have a multi-task learning problem with *t* tasks, where each task $i \in \{1, \cdots , t\}$ is associated with a set of samples $(X_i, \varvec{y_i}), X_i \in R^{n_i \times p}, \varvec{y_i} \in R^{n_i}$. We denote $X = [X_1, \cdots , X_t], Y = [\varvec{y_1}, \cdots , \varvec{y_t}]$ and $W = [\varvec{w_1}, \cdots , \varvec{w_t}] \in R^{p\times t}$ represents the coefficient matrix over all tasks. Referring to Fig.  1, the *k*-th task corresponds to the prediction on *k*-th time point. To learn the *t* tasks simultaneously, the following regularized empirical risk is minimized:

$$\begin{aligned} \min _W {\mathcal {L}}(W) + \varvec{\Omega } (W), \end{aligned}$$

where ${\mathcal {L}}(W)$ denotes the loss function and $\varvec{\Omega } (W)$ is the regularization term that encodes the prior knowledge.

### Adaptive global temporal structure

In our model, total *t* tasks correspond to *t* time points. We assume the *k*-th time point is related to all previous time points, meaning the *k*-th task $\varvec{w_k}$ is related to all previous tasks $\varvec{w_i}, i = [1, \cdots , k-1]$. We use matrix multiplication to model this idea, enabling our model to share information among correlated tasks. Before showing details of our method, we first give a new definition termed “temporal task”, denoted as $\varvec{{\bar{\omega}}}$.

#### Definition 1

The *i*-th temporal task $\varvec{{\bar{\omega}}_i}$ satisfies

$$\begin{aligned} \left\{ \begin{aligned}&\varvec{ {\bar{\omega}}_1} = \varvec{w_1} \\&{\varvec{ {\bar{\omega}}_2}} = \alpha \varvec{ {\bar{\omega}}_1} + (1-\alpha ) \varvec{ w_2} \\&\cdots \\&{\varvec{ {\bar{\omega}}_t}} = \alpha \varvec{ {\bar{\omega}}_{t-1}} + (1-\alpha ) \varvec{ w_t}. \end{aligned} \right. \end{aligned}$$

In the above Definition 1, the parameter $\alpha \in [0, 0.5]$ represents the relational degree between the current *i*-th time point and all previous time points. The upper bound of $\alpha$ we set $\frac{1}{2}$, means the state at the current time point is more important than previous states, which corresponds with reality to a certain extent. Actually, the value of $\alpha$ depends on the result of cross-validation, i.e., we can adaptively capture the global temporal relation among multiple time points (tasks).

Now according to (1), we formulate this kind of relation via the following matrix multiplication:

1 $$\begin{aligned} W {\mathfrak {R}}(\alpha ) = W A_1(\alpha )A_2(\alpha )\cdots A_{t-1}(\alpha ), \end{aligned}$$

where the matrix $$ {\mathfrak {R}}(\alpha )$$, representing the correlation among tasks, is a function of the hyperparameter $$\alpha $$; $$ A_i(\alpha ) \in R^{t \times t}$$ is an identity matrix, and the value of $$A_{i}(\alpha )_{m,n}$$ is replaced by $$\alpha $$ if $$ m=i,n=i+1$$, the value of $$A_{i}(\alpha )_{m,n}$$ is replaced by $$(1-\alpha )$$ if $$ m=n=i+1$$. The following adaptive global temporal structure (AGTS) (2) is the expanding form of (1).

2 $$\begin{aligned} W {\mathfrak {R}}(\alpha ) = W \begin{bmatrix} 1&{}\alpha &{}\cdots &{}0\\ 0&{}1-\alpha &{}\cdots &{}0\\ \vdots &{} \vdots &{} \ddots &{} \vdots \\ 0&{} 0&{} \cdots &{} 1 \end{bmatrix} \cdots \begin{bmatrix} 1&{} 0&{}\cdots &{}0\\ \vdots &{} \vdots &{} \ddots &{} \vdots \\ 0&{} 0&{}\cdots &{}\alpha \\ 0&{} 0&{} \cdots &{} 1-\alpha \end{bmatrix}. \end{aligned}$$

It is worth noting that ${\mathfrak {R}}(\alpha )$ is an upper triangular and full rank matrix, meaning the invertibility of ${\mathfrak {R}}(\alpha )$. This property demonstrates the difference between our AGTS and existing low-rank approaches, including the methods with trace norm [1, 14, 30] and task grouping with latent basis task methods [2, 23, 42]. We emphasize this property is significant for designing the efficient AGM-based optimization algorithm, shown in Sect.  4, associated with our proposed novel formulation. Lemma 1 gives a deeper understanding of the AGTS mechanism, related to the concepts of convex hull [4] and non-decreasing order.

#### Lemma 1

For any i $\in \{1, \cdots , t\}$, $\varvec{r_i} = [r_i^1, \cdots , r_i^i, 0, \cdots , 0]^T\in R^t$ is *i*-th column of satisfies

3 $$\begin{aligned}{} & {} \sum \nolimits _{k=1}^{i} r_i^k = 1, \end{aligned}$$

4 $$\begin{aligned}{} & {} 0 \leqslant r_i^1 \leqslant r_i^2 \cdots \leqslant r_i^i. \end{aligned}$$

#### Proof

Denote $R = R(\alpha )$, $\varvec{e_i} \in R^t$ is an identity vector whose *i*-th entry is 1. According to (1), for any $i \in \{2, \cdots , t\}$, we have

5 $$\begin{aligned} \left\{ \begin{aligned}&\varvec{r_1} = \varvec{e_1}, \\&\varvec{r_i} = \alpha \varvec{r_{i-1}} + (1-\alpha ) \varvec{e_i}. \end{aligned} \right. \end{aligned}$$

It is clear that $\sum \nolimits _{k=1}^{1} {r}_1^k = 0 \Rightarrow \sum \nolimits _{k=1}^{i-1} {r}_{i-1}^k = 0 \Rightarrow \sum \nolimits _{k=1}^{i}r_i^k = 1$, that results in (3). Since $\alpha \in [0, 0.5]$, for $\varvec{r_2}$, $0 \leqslant r_2^1 = \alpha \leqslant r_2^2 = (1-\alpha )$. By mathematical induction, we assume $\varvec{r_{m-1}}$ satisfies (4), $\varvec{r_m} = \alpha \varvec{r_{m-1}} + (1-\alpha ) \varvec{e_m}$, so $\sum \nolimits _{k=1}^{m-1}r_m^k = \alpha \leqslant r_m^m = (1-\alpha )$, we have (4). It completes the proof. $\square$

Lemma  1 tells the two characteristics of AGTS (2):

- $\varvec{{\bar{\omega}}_k}$ is a convex hull [4], a kind of special linear combination, of $\{\varvec{\bar{\omega}_1}, \cdots , {\varvec{\bar{\omega}}}_k\}$. It means we consider all the time points from time point 1 to *k*, that is the reason why we call it the global temporal structure.
- The non-decreasing order of the entry of ${\varvec{r}}$ means the farther the distance, the less the impact. Specifically, the farther away the time point is from the current time point, the less influence it has on the current time point, which is in line with general practical problems.

### Temporal sparse group lasso

We want our approach to have the ability to conduct feature selection such that the selected important features are usually meaningful in many scenarios like bioinformatics, medicine, chemistry, etc. The Lasso penalty [37] is one of the most commonly used penalties since it introduces sparsity into the model. Group Lasso penalty [44] is an extension of Lasso, considering the natural grouping of features. The combination of Lasso and group Lasso penalties is also known as the sparse group Lasso penalty [36], which allows simultaneous joint feature selection for all tasks and the selection of a specific set of features for each task. However, the sparse group Lasso treats every task equally without considering the complex correlation of tasks. We combine our AGTS with sparse group Lasso to propose a temporal sparse group Lasso which considers the global temporal relatedness among tasks and conducts feature selection in the meantime. After denoting ${\mathfrak {R}}(\alpha ) = {\mathfrak {R}}$ to lighten notation, the proposed temporal sparse group Lasso penalty can be mathematically denoted as

$$\begin{aligned} \Vert W{\mathfrak {R}}\Vert _1 + \Vert W{\mathfrak {R}}\Vert _{1,2}. \end{aligned}$$

where $\Vert W{\mathfrak {R}} \Vert _1$ is the Lasso penalty of $(W{\mathfrak {R}})$, the group Lasso penalty $\Vert W{\mathfrak {R}} \Vert _{1,2}$ is given by $\sum _{i=1}^{p}\sqrt{\sum _{j=1}^{t}(W{\mathfrak {R}})_{i,j}^2}$.

### Global temporal smoothness

Existing MTL methods based on temporal smoothness [10, 35, 40, 47, 50, 54] have achieved great success, in which every time point corresponds to a prediction task. Based on the regression model, they assume the difference of the predictions between successive time points is small. However, the possible limitation is this assumption only focuses on the adjacent time points without considering the complex correlation among multiple time points, i.e., only chases the local temporal smoothness assumption. We combine this assumption with our AGTS to propose two novel penalties, mathematically denoted as

$$\begin{aligned} \Vert W{\mathfrak {R}}H \Vert _F^2 \text { and } \Vert (W{\mathfrak {R}}H)^T \Vert _1, \end{aligned}$$

where the matrix H $\in R^{t\times (t-1)}$ is a sparse matrix in which $H_{i,i} = 1$ and $H_{i,i+1} = -1$. Since AGTS takes into account all previous time points of the current time point, rather than only the successive time point, we call this the global temporal smoothness assumption.

The difference between the two penalties is the first $\Vert W{\mathfrak {R}}H \Vert _F^2$, termed global Laplacian-based smoothness penalty, focuses on the smoothness of the prediction models across different time points, while the second $\Vert (W{\mathfrak {R}}H)^T\Vert _1$, named global fused Lasso based smoothness penalty, enforces the selected features across different time points are smooth. Thus the latter penalty better captures the global temporal smoothness of selected features, which is closer to the real-world progression mechanism. Another reason is although the use of the Laplacian-based smoothness penalty can avoid the computational difficulty, we show in Sect.  4 that the novel framework with the global fused Lasso based smoothness penalty also can be solved efficiently.

### Adaptive temporal multi-task learning

We combine the temporal sparse group Lasso with the global fused Lasso based smoothness penalty to propose a novel multi-task formulation, termed adaptive temporal multi-task learning (ATMTL), and mathematically denoted as

6 $$\begin{aligned} \min _{W}{\mathcal {L}}(W) + \lambda _1\Vert W{\mathfrak {R}} \Vert _1 + \lambda _2\Vert W{\mathfrak {R}}\Vert _{1,2} + \lambda _3\Vert (W{\mathfrak {R}}H)^T \Vert _1, \end{aligned}$$

where ${\mathcal {L}}(W)$ is the empirical loss function, which becomes a squared loss $\sum \nolimits _{i=1}^{t}\Vert X\varvec{w_i} - \varvec{y_i}\Vert _2^2$ for regression problem and a logistic loss $\sum \nolimits _{i=1}^{t} \sum \nolimits _{j = 1}^{n_i} \log (1 + \exp (-y_j^nX_i^j\varvec{w_i}))$ for binary classification problem; ${\mathfrak {R}} = {\mathfrak {R}}(\alpha )$, and $\lambda _1$, $\lambda _2$, $\lambda _3, \alpha$ are fine-tuned parameters. It is clear that ${\mathfrak {R}}(\alpha )$ can adaptively capture the global temporal relatedness among tasks. The temporal sparse group Lasso $\lambda _1\Vert W{\mathfrak {R}} \Vert _1 + \lambda _2\Vert W{\mathfrak {R}}\Vert _{1,2}$ is used to perform feature selection at both group level and within group level. The global temporal smoothing penalty $\Vert (W{\mathfrak {R}}H)^T \Vert _1$ enforces the state of the system does not fluctuate drastically over time.

## Optimization algorithm

In this section, we give the details of the two associated optimization algorithms, the ADMM-based algorithm, and the AGM-based algorithm.

### The ADMM-based algorithm

In recent years, the alternating direction method of multipliers (ADMM) [5] has attracted much attention, since it is easy to parallelize distributed convex problems. In ADMM, the global optimal solution is determined by coordinating the solutions of local subproblems.

The original Eq. (6) is equivalent to the following constrained problem:

7 $$\begin{aligned}& \min _{W,A,B} L(W) + \lambda _1\Vert A \Vert _1 + \lambda _2 \Vert A \Vert _{1,2} + \lambda _3 \Vert B\Vert _{1}, \nonumber \\ & \quad \text {s.t. } W{\mathfrak {R}} = A, W{\mathfrak {R}}H = B, \end{aligned}$$

where *A*, *B* are auxiliary variables. Note that we use only one auxiliary matrix A to relax both the Lasso penalty and group Lasso penalty to reduce the computational complexity. The augmented Lagrangian function of (7) is

8 $$\begin{aligned} L_\rho&{(W,A,B,C,D)} = \frac{1}{2}\Vert XW-Y\Vert _F^2 \nonumber \\&+ \lambda _1 \Vert A\Vert _1 + \lambda _2\Vert A\Vert _{1,2} +\lambda _3 \Vert B\Vert _1\nonumber \\&+ Tr(C^T(W{\mathfrak {R}}-A)) + \frac{\rho }{2}\Vert WR{\mathfrak {R}}-A\Vert _F^2 \nonumber \\&+ Tr(D^T(W{\mathfrak {R}}H-B))+\frac{\rho }{2}\Vert W{\mathfrak {R}}H-B\Vert _F^2. \end{aligned}$$

#### Update W

For the regression problem with a squared loss, we use inexact ADMM [17, 29], which is shown to have the same convergence rate as exact updates [5], to improve efficiency. From the augmented Lagrangian in (8), the update of *W* is carried out by setting the gradient of *W* to 0, we have

9 $$\begin{aligned}&\sum \nolimits _{i=1}^{t} X_i^T(X_iw_i - y_i) + \rho WE + \rho WF\\ \nonumber&\quad = \rho G + \rho K-L-J+ X^TY, \end{aligned}$$

where $E = {\mathfrak {R}}{\mathfrak {R}}^T, N = {\mathfrak {R}}H, F = NN^T, G = A{\mathfrak {R}}^T, K = BN^T, L = C{\mathfrak {R}}^T, J = DN^T$. Clearly, we find that the columns of *W* are coupled, which makes the directed update of *W* is difficult. Now we show the update of *W* can be conducted in an efficient way using a suitable linearization method. To be specific, for $(K+1)$-th iteration, we have

10 $$\begin{aligned} V_i\varvec{w_i^{k+1}}&=\varvec{q_i^{k}}, i \in \{1, \cdots , t\}. \end{aligned}$$

11 $$\begin{aligned} V_i&=X_{i}^TX_{i} + \rho (1 + M_{ii})I_{p\times p}. \end{aligned}$$

12 $$\begin{aligned} \varvec{q_i^{k}}&= X_{i}^T\varvec{y_i} - \varvec{l_i^{k}} + \rho \varvec{g_i^k} - \varvec{j_i^k} + \rho \varvec{k_i^k} \\ \nonumber&\quad - \rho \sum \nolimits _{j=1, j\ne i}^{t}\varvec{w_j^k} M_{ji}. \end{aligned}$$

where $M = E+F$. It is clear that $V_i, i \in \{1, \cdots , t\}$ is symmetric positive definite, which Cholesky factorization is applicable for, resulting in efficient updating of *W*.

For binary classification problems with a logistic loss, it is solved by using L-BFGS [34], where the gradient is

$$\begin{aligned} \nabla _{\varvec{w_i}} =&- \frac{1}{n_i}\sum \nolimits _{j= 1}^{n_i} \frac{\exp (-y_i^j(X_i^j \varvec{w_i}))}{1 + \exp (-y_j^i(X_i^j \varvec{w_i}))}y_i^j (X_i^j)^T \\&+ \varvec{l_i} + \varvec{y_i} + \rho (W{\mathfrak {R}}-A)\varvec{s_i} + \rho (WN-B)\varvec{u_i} \end{aligned}$$

where $$S = {\mathfrak {R}}^T$$, $$U = N^T$$.

#### Update auxiliary variables

We need to update the two auxiliary variables *A* and *B* at *k*-th iteration. And the corresponding minimization problems are

13 $$\begin{aligned} A^{k+1}&= \arg \min _{A}\frac{1}{2}\Vert A-\left(W^{k+1}{\mathfrak {R}}+\frac{C^{k}}{\rho }\right)\Vert _F^2 \\ \nonumber{} & {} \quad +\frac{1}{\rho }(\lambda _1\Vert A\Vert _1 + \lambda _2\Vert A\Vert _{1,2}), \end{aligned}$$

14 $$\begin{aligned} B^{k+1}&= \arg \min _{B}\frac{1}{2}\Vert B-\left(W^{k+1}{\mathfrak {R}}H+\frac{D^{k}}{\rho }\right)\Vert _F^2 + \frac{\lambda _3}{\rho }\Vert B\Vert _1 . \end{aligned}$$

According to [43], (13) has an analytical solution with decoupling each row of matrix A. We introduce the following two lemmas to solve (13) and efficiently.

##### Lemma 2

[28] For any $$\lambda \geqslant 0$$,

$$\begin{aligned} \pi ({\varvec{v}})&= \arg \min _{{\varvec{w}}} \frac{1}{2}\Vert {\varvec{w}}-{\varvec{v}}\Vert _2^2 + \lambda \Vert {\varvec{w}}\Vert _2 \\&= \max \{\Vert {\varvec{v}}\Vert _2-\lambda , 0\} \frac{{\varvec{v}}}{\Vert {\varvec{v}}\Vert _2}. \end{aligned}$$

##### Lemma 3

[43, 53] For any $$\lambda _1, \lambda _2$$,

$$\begin{aligned}&\pi _\textrm{Lasso}({\varvec{v}}) = \arg \min _{{\varvec{w}}}\frac{1}{2}\Vert {\varvec{w}}-{\varvec{v}}\Vert _2^2 + \lambda _1\Vert {\varvec{w}}\Vert _1. \\&\pi _\textrm{GLasso}({\varvec{v}}) = \arg \min _{{\varvec{w}}}\frac{1}{2}\Vert {\varvec{w}}-{\varvec{v}}\Vert _2^2 + \lambda _2\Vert {\varvec{w}}\Vert _2.\\&\pi ({\varvec{v}}) = \arg \min _{{\varvec{w}}}\frac{1}{2}\Vert {\varvec{w}}-{\varvec{v}}\Vert _2^2 + \lambda _1\Vert {\varvec{w}}\Vert _1 + \lambda _2\Vert {\varvec{w}}\Vert _2. \end{aligned}$$

Then the following holds:

$$\begin{aligned} \pi ({\varvec{v}}) = \pi _\textrm{GLasso}(\pi _\textrm{Lasso}({\varvec{v}})). \end{aligned}$$

Note that, as for solving (14), we decouple each column of B, since we chase the global temporal correlation among multiple time points.

#### Update dual variables

Following standard ADMM dual update [5], the update for dual variable for our setting is as follows:

15 $$\begin{aligned}&C^{k+1} = C^{k} + \rho (W^{k+1}{\mathfrak {R}}-A^{k+1}), \end{aligned}$$

16 $$\begin{aligned}&D^{k+1} = D^{k} + \rho (W^{k+1}{\mathfrak {R}}H-B^{k+1}). \end{aligned}$$

#### The stopping criteria

We need to compute the primal and dual residual, which can be considered as the stopping criteria. For the problem (8), the primal residual and dual residual are

$$\begin{aligned} P^{k+1} = \Vert W^{k+1}{\mathfrak {R}}-A^{k+1}\Vert _F + \Vert W^{k+1}{\mathfrak {R}}H - B^{k+1}\Vert _F, \\ S^{k+1}= \Vert \rho (A^{k+1}-A^k)+ \rho (B^{k+1}-B^k)H^T\Vert _F. \end{aligned}$$

The stopping criterion is both $P^{k+1}$ and $S^{k+1}$ are relatively small.

Algorithm 1 summarizes the whole procedure.
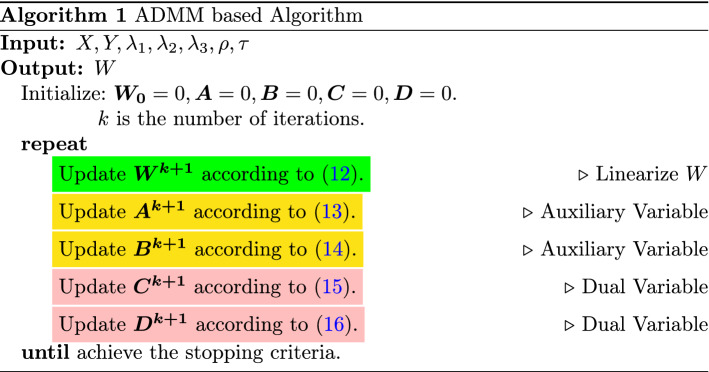


#### Convergence rate

Although ADMM is widely used in the MTL community, the convergence rate of ADMM is only ${\mathcal {O}}(1/k)$ for k iterations [16] and the worst-case convergence rate is ${\mathcal {O}}(1/\sqrt{k})$ which is quite slow [43]. More than that, the actual speed of the implementation of ADMM may rely on the choice of the penalty parameter $\rho$. It is challenging to design an ADMM-based algorithm with high efficiency. So we additionally devise an efficient algorithm based on AGM and the special structure of our formulation.

### The efficient AGM-based algorithm

Due to the optimal convergence rate for the class of first-order methods, i.e., ${\mathcal {O}}({1}/{k^2})$ for k iterations, the accelerated gradient method (AGM) [32] has been extensively utilized to solve multi-task learning problems of the following form:

17 $$\begin{aligned} \min _{W}F(W) = f(W) + g(W), \end{aligned}$$

where *f*(*W*) is convex and smooth, *g*(*W*) is convex but nonsmooth. The AGM is based on two sequences, the approximation point $\{W_i\}$ and the search point $\{ S_i\}$. ${S_i}$ is the affine combination of $W_{i-1}$ and $W_i$, denoted as

$$\begin{aligned} S_{i+1} = W_i + \alpha _i(W_i - W_{i-1}), \end{aligned}$$

where $\alpha _i$ is the combination coefficient. Following the strategy in [3], we set $\alpha _i = \frac{(t_{i-1} - 1)}{t_i}$, $t_0 = 1$ and $t_i = \frac{1}{2}(1+\sqrt{4t_{i-1}^2+1})$ for $i \geqslant 1$.

The approximate solution $W_i$ is computed as $W_i = \pi (S_i-\frac{1}{L}f^\prime (S_i))$, where the notation $\pi (V)$ is the proximal operator of *V*, $\frac{1}{L}$ is the stepsize, which is important for the global convergence of the accelerated gradient-based algorithms. The stepsize 1/*L* can be estimated with many sophisticated line-search schemes [4] in general. Specifically, the value of *L* is updated until satisfying

18 $$\begin{aligned} f(W_i) \leqslant f(S_i) + \langle \nabla f(S_i), W_i - S_i \rangle + \frac{L}{2}\Vert W_i - S_i\Vert _F^2. \end{aligned}$$

However, this updating procedure may incur overhead costs in the computation, especially in the case where the dimension of dataset is very large, i.e., several million [3].

#### Estimation of the Lipschitz constant

To avoid the expensive computational cost of estimating the Lipschitz Constant for *f*(*W*), in the case of regression problem, we can directly compute its best value (the smallest Lipschitz constant) as summarized in the following lemma.

##### Lemma 4

Given $X = [X_1, \cdots , X_t], X_i \in R^{n_i \times p}, Y = [\varvec{y_1}, \cdots , \varvec{y_t}]$, $\varvec{y_i} \in R^{n_i}$. The best Lipschitz constant $L_f$ of the function *f*(*W*) is no larger than $\sigma _X^2$, where $\sigma _X = \max \{\sigma _{X_i}\}, i \in \{1, \cdots , t\}$, $\sigma _{X_i}$ is the largest singular value of $X_i$.

##### Proof

This proof is similar to [8], which however only considers the scenario all tasks have the same samples. We extend it to tasks with different samples. $\square$

Algorithm 2 summarizes the whole procedure.
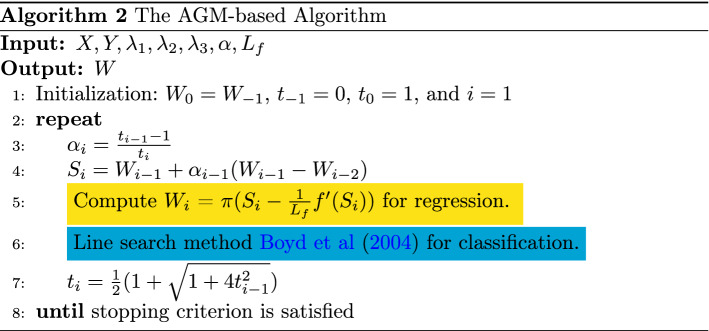


#### Compute the proximal operator

For designing an efficient AGM-based algorithm, the most pivotal step is computing the proximal operator of three non-smooth penalties in (6). We show that based on the special structure of our formulation, it can be done in an efficient way. Note that $\mathfrak {R(\alpha )}$ is a full rank matrix that is invertible, we denote $W{\mathfrak {R}} = {\mathcal {Q}}$, ${\mathcal {S}} = {\mathfrak {R}}^{-1}$, so we transfer (6) to the following form:

19 $$\begin{aligned} \min _{W}{\mathcal {L}}(\mathcal {Q, S}) + \lambda _1\Vert {\mathcal {Q}} \Vert _1 + \lambda _2\Vert {\mathcal {Q}}\Vert _{1,2} + \lambda _3\Vert ({\mathcal {Q}}H)^T \Vert _1. \end{aligned}$$

Since the matrix ${\mathcal {S}}$ depends on cross-validation, (19) is convex, which guarantees us to achieve the global minimum easily.

The proximal operator of three penalties in (19) is

20 $$\begin{aligned} \pi (V) = \arg \min _{{\mathcal {Q}}}\frac{1}{2}\Vert {\mathcal {Q}}-V\Vert _F^2 + \lambda _1\Vert {\mathcal {Q}} \Vert _1 + \lambda _2\Vert {\mathcal {Q}}\Vert _{1,2} + \lambda _3\Vert F{\mathcal {Q}}^T \Vert _{1}, \end{aligned}$$

where $F = H^T$. It is clear that each row of ${\mathcal {Q}}$ is decoupled in (20). Thus for obtaining ${\varvec{q}}$, the row vector of ${\mathcal {Q}}$, we need to solve

21 $$\begin{aligned} \pi ({\varvec{v}}) = \arg \min _{{\varvec{q}}}\frac{1}{2}\Vert {\varvec{q}}-{\varvec{v}}\Vert _2^2 + \lambda _1\Vert {\varvec{q}} \Vert _1 +\lambda _2\Vert {\varvec{q}} \Vert _2+ \lambda _3\Vert F{\varvec{q}} \Vert _{1}, \end{aligned}$$

where ${\varvec{v}}$ is the row vector of V. The solution process of (21) has the certain decomposition property according to [52], so (21) can be solved efficiently. The specific procedure is shown as follows:

22 $$\begin{aligned}{} & {} \pi _{FL}({\varvec{v}}) = \arg \min _{{\varvec{q}}}\frac{1}{2}\Vert {\varvec{q}}-{\varvec{v}}\Vert _2^2 + \lambda _1\Vert {\varvec{q}} \Vert _1 + \lambda _3\Vert F{\varvec{q}} \Vert _{1},\nonumber \\{} & {} \pi _{GL}({\varvec{v}}) = \arg \min _{{\varvec{q}}}\frac{1}{2}\Vert {\varvec{q}}-{\varvec{v}}\Vert _2^2 + \lambda _2\Vert {\varvec{q}} \Vert _2, \end{aligned}$$

23 $$\begin{aligned}{} & {} \Rightarrow \pi ({\varvec{v}}) = \pi _{GL}(\pi _{FL}({\varvec{v}})). \end{aligned}$$

The proximal operator of fused Lasso () can be effectively solved using [27] and (22,23) has an analytical solution according to [28], so we can solve (21) with high efficiency.

## Experiment

In this section, we first introduce four real-world dataset used in this paper. The difference of the performance of our ADMM-based and AGM-based algorithms on regression and classification problems is shown in Sect. 5.2.We choose the better AGM-based algorithm to compete with several MTL methods that consider the task relation in terms of efficiency. For evaluating the effectiveness, we conduct comprehensive experiments comparing several recently proposed MTL approaches on different datasets. The implementation code of the method is on Matlab and can be found at https://github.com/menghui-zhou/ATMTL. The processor is Intel i5 6500, CPU 2.5GHz.

To be specific, we compare the efficiency for the regression problem of the ADMM-based algorithm and the AGM-based algorithm on the ADAS dataset, and for the classification problem on the Employee dataset. We also compare the efficiency between our ATMTL and several baseline methods on the MMSE dataset. After this, we evaluate the effectiveness of our proposed temporal sparse group Lasso on the Parkinson dataset, and our proposed global temporal smoothness on the MMSE dataset. Finally, we demonstrate the effectiveness of our ATMTL on the COVID-19 dataset for regression problems and the Employee dataset for the binary classification problem, compared with several baseline methods.

**Table 1:** Details of the dataset. The value of ‘Type’ column, R means regression problem, and C means classification problem

Name	Type	Feature number	Task number
ADAS	R	314	6
MMSE	R	314	6
Parkinson	R	18	4
Covid-19	R	10	4
Employee	C	20	10

**Table 2:** The detailed information of sample numbers at different time points of AD dataset

Dataset	M00	M06	M12	M24	M36	M48
MMSE	1092	1078	1027	883	579	494
ADAS	1074	1064	1014	867	556	483

There are total 6 time points. In this table, the sample size indicates the number of patients that has baseline MRI features and corresponding target cognitive scores (MMSE or ADAS) at future time points

### Dataset

In this subsection, we briefly introduce the information of the dataset used in this paper.

- **Parkinson dataset** [39]: This dataset is composed of a range of biomedical voice measurements from 42 people with early-stage Parkinson’s disease recruited to a six-month trial of a telemonitoring device for remote symptom progression monitoring. The recordings were automatically captured in the patient’s homes. The goal is to predict the Unified Parkinson’s Disease Rating Scale (UPDRS) score for each patient according to their 16 biomedical features. Every thirty days as a period, we calculate the average UPDRS score for each period. Finally, we choose the first four months as the four time points corresponding to four regression tasks.
- **Alzheimer’s disease (AD) dataset** [20]: In order to better understand the disease, NIH in 2003 founded the Alzheimer’s Disease Neuroimaging Initiative (ADNI) to facilitate the scientific evaluation of positron emission tomography (PET), magnetic resonance imaging (MRI), and other biomarkers. This big dataset is used to predict the cognitive scores, including the AD Assessment Scale-Cognitive Subscale (ADAS-Cog, ADAS) and the Mini-Mental State Exam (MMSE), of AD patients at consecutive time points (6-month or 1-year intervals). In this study, we have 314 MRI features and 6 time points, from baseline time point (M00) to M48, meaning 48 months away from baseline time points. Every time point stands for a regression task.
- **Covid-19 dataset** [9, 15]: This COVID-19 dataset we have processed consists of two datasets. The first dataset is the real-time number of COVID-19 patients in different regions of the world [9].The second is the quantitative data of specific COVID-19 policies of each country processed by [15].We combine these two datasets to predict the number of COVID-19 cases for four future weeks. Each week is viewed as a time point as well as a regression task. Finally, we have the data of 50 countries, including China, the UK, the USA, Canada, and so on. There are 10 features, including population and density of population.
- **Employee attrition dataset**^1^ The employee attrition dataset provided by IBM Waston Analytics is also used to evaluate the performance of our approach for binary classification problem. We study the problem of whether the employees are still working in the company in the *k*-th year since they joined the company. We consider this problem within ten years corresponding to 10 time points. Every time point is considered as a binary classification task. There are 20 features in this dataset, including years at the company: How many years has the employee stayed at the company before leaving? years with current manager: how many years has the employee stayed in the current role, and so on.

Table 1 shows the details of our used dataset. Since the samples of the AD dataset are different, we put the detailed information of the AD dataset in Table 2.

### Efficiency

In this subsection, we first compare the performance of our ADMM-based and AGM-based algorithms in detail and then show the experimental results of the comparison with several baseline MTL methods that take into account the task relation.

**Fig. 2:**
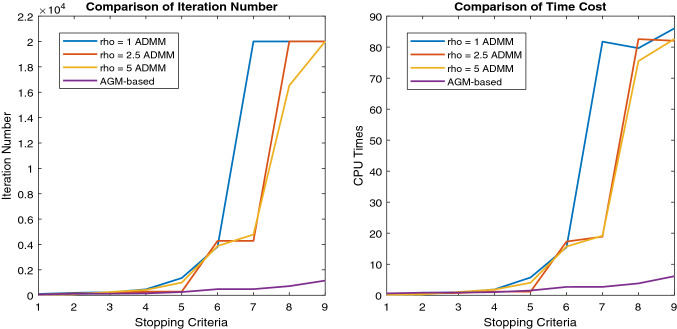
Comparison of the efficiency of the ADMM-based algorithm and the AGM-based algorithm on ADAS dataset. The stopping criterion is from $10^1$ to $10^{-7}$. If the difference of the function value of two consecutive iterations is less than the stopping criterion, we terminate the program

**Fig. 3:**
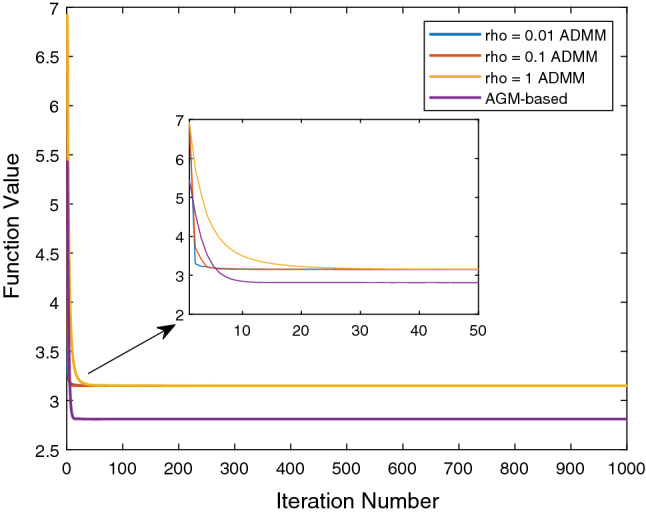
Comparison of the convergence situation of the ADMM-based algorithm and the AGM-based algorithm on Employee dataset. The maximum iteration number is 1000. When the number of iterations is up to 1000, we terminate the program

**Table 3:** Comparison of CPU time (s) of algorithms with different maximum iteration number on Employee dataset

Iteration number	$\rho =0.01$	$\rho =0.1$	$\rho =1$	AGM
10	0.213	0.225	0.208	**0**.**083**
100	19.9	16.4	12.7	**0**.**721**
1000	94.0	26.5	84.7	**6**.**92**

Bold number indicates the best performance, i.e., the lowest nMSE

**Table 4:** The range of the hyper parameter of the involved feature selection methods

Method	Parameter range
Lasso	$\lambda \in [10^{-3}, \cdots , 10^4]$
gLasso	$\lambda \in [10^{-3}, \cdots , 10^4]$
hLasso	$\lambda \in [10^{-3}, \cdots , 10^4]$
gBridge	$\lambda \in [10^{-3}, \cdots , 10^4],$
	$$\gamma \in [0.1, 0.25, 0.5, 0.7, 0.8, 0.9]$$
sgLasso	$\lambda _1, \lambda _2 \in [10^{-3}, \cdots , 10^4]$
LES	$\lambda \in [10^{-3}, \cdots , 10^4],$
	$$ \alpha \in [0.1, 1, 2, e, 5, 10]$$
**tsgLasso ** $\star$	$\lambda _1, \lambda _2 \in [10^{-3}, \cdots , 10^4]$
	$$\alpha \in [0.01, 0.02, 0.05, 0.1, \cdots , 0.5]$$

**Table 5:** Comparison on Parkinson dataset of feature selection methods in terms of the mean value of nMSE (mean) and standard deviation (std)

Task No	Lasso	gLasso	hLasso	gBridge	spLasso	LES	**tsgLasso **$\star$
2	0.933 ± 0.053	0.927 ± 0.083	1.131 ± 0.240	0.926 ± 0.049	0.918 ± 0.067	0.935 ± 0.043	**0**.**909** ± **0**.**052**
3	1.023 ± 0.200	0.965 ± 0.202	1.265 ± 0.383	0.955 ± 0.137	0.952 ± 0.197	0.988 ± 0.154	**0**.**948** ± **0**.**201**
4	1.007 ± 0.057	0.958 ± 0.068	1.244 ± 0.202	0.967 ± 0.082	**0**.**948** ± **0**.**069**	0.972 ± 0.103	**0**.**948** ± **0**.**057**

Bold number indicates the best performance, i.e., the lowest nMSE

The symbol $\star$ means our approach

**Table 6:** The range of the hyperparameter of the involved temporal smoothness penalties

Method	Parameter range
Lbased	$\lambda \in [10^{-3}, \cdots , 10^4]$
FLbased	$\lambda \in [10^{-3}, \cdots , 10^4]$
GLbased	$\lambda \in [10^{-3}, \cdots , 10^4]$
	$$\alpha \in [0.01, 0.05, 0.1, \cdots , 0.5]$$
GFLbased $\star$	$\lambda \in [10^{-3}, 10^{-2}, \cdots , 10^4]$
	$$\alpha \in [0.01, 0.02, 0.05, 0.1, \cdots , 0.5]$$

**Table 7:** Comparison on MMSE dataset of temporal smoothness penalties in terms of the mean value of nMSE (mean) and standard deviation (std). The symbol $\star$ means our approach

Task number	Lbased	FLbased	GLbased	GFLbased $\star$
2	0.700 ± 0.054	0.686 ± 0.053	0.696 ± 0.051	**0**.**678** ± **0**.**049**
4	0.640 ± 0.038	0.626 ± 0.036	0.638 ± 0.039	**0**.**622** ± **0**.**033**
6	0.601 ± 0.028	**0**.**582** ± **0**.**029**	0.599 ± 0.029	0.583 ± 0.027

Bold number indicates the best performance, i.e., the lowest nMSE

**Table 8:** Detailed information of several baseline multi-task learning methods, including the hyper parameter range

Model	Name	Penalty	Parameter range
$W = P + U$	RMTL [7]	$\lambda _1\Vert P\Vert _{*} + \lambda _2\Vert U\Vert _{1,2}$	$\lambda _1, \lambda _2 \in [10^{-3}, \cdots , 10^4]$
	MNorm [8]	$\lambda _1\Vert P\Vert _{*} + \lambda _2\Vert U\Vert _{1}$	$\lambda _1, \lambda _2 \in [10^{-3}, \cdots , 10^4]$
$W = PU$	LTGO [23]	$\lambda _1\Vert P\Vert _{1} + \lambda _2\Vert U\Vert _{F}^2$	$\lambda _1, \lambda _2 \in [10^{-3}, \cdots , 10^4]$
			$$k \in [1, t/3, t/2, 2t/3,t]$$
	VSTG [22]	$\lambda _1\Vert P\Vert _{1} + \lambda _2\Vert P\Vert _{1,\infty } + \lambda _3\sum _{i=1}^{t}(\Vert \varvec{u_i}\Vert _k^{sp})^2$	$\lambda _1, \lambda _2, \lambda _3 \in [10^{-3}, \cdots , 10^4]$
Temporal	TGL [51]	$\lambda _1 \Vert W\Vert _F^2+\lambda _2 \Vert WH\Vert _F^2+\lambda _3 \Vert W\Vert _{1,2}$	$\lambda _1, \lambda _2 \in [10^{-3}, \cdots , 10^4]$
	cFSGL [52]	$\lambda _1 \Vert W\Vert _1+\lambda _2 \Vert FW^T\Vert _1+\lambda _3 \Vert W\Vert _{1,2}$	$\lambda _1, \lambda _2 \in [10^{-3}, \cdots , 10^4]$
Calibrated	MTFLC [13]	$\lambda _1 \Vert W\Vert _{1,2} + \lambda _2 \Vert W\Vert _F^2$	$\lambda _1, \lambda _2 \in [10^{-3}, \cdots , 10^4]$
	NC-CMTL [33]	$\mu \sum _{i=1}^{p}\log (\sigma _i(W) + 1)$	$\lambda \in [10^{-3}, \cdots , 10^4]$
Ours$\star$	ATMTL	$\lambda _1 \Vert W{\mathfrak {R}}\Vert _1+\lambda _2 \Vert W{\mathfrak {R}}\Vert _{1,2}+\lambda _3 \Vert (W{\mathfrak {R}}H)^T\Vert _{1}$	$\lambda _1, \lambda _2, \lambda _3 \in [10^{-3}, \cdots , 10^4]$
			$$\alpha \in [0.01, 0.02, 0.05, 0.1, \cdots , 0.5]$$

**Table 9:** Comparison with several baseline methods on COVID-19 dataset with the setting of different task number. For regression problem, evaluate the performance of all methods in term of nMSE (mean ± std). The symbol $\star$ means our approach

Task Number	nMSE	RMTL	MNorm	LTGO	VSTG	TGL	cFSGL	MTFLC	NC-CMTL	ATMTL$\star$
2	mean std	1.183	1.009	1.247	1.034	0.995	0.828	1.228	1.254	**0**.**793**
		0.254	0.133	0.448	0.395	0.327	0.224	0.199	0.503	0.198
3	mean std	1.159	1.130	0.992	1.008	0.717	**0**.**713**	0.858	1.168	0.714
		0.439	0.211	0.315	0.354	0.161	0.165	0.175	0.302	0.166
4	mean std	0.960	0.721	0.928	0.951	0.646	0.642	0.681	0.926	**0**.**638**
		0.099	0.144	0.163	0.159	0.196	0.098	0.192	0.196	0.107

Bold number indicates the best performance, i.e., the lowest nMSE

**Table 10:** Comparison with several baseline methods on Employee dataset with the setting of different training ratio. For binary classification problem, evaluate the performance of all methods in term of ACC. The symbol $\star$ means our approach

Ratio	DMTL	rMTFL	LTGO	VSTG	TGL	cFSGL	ATMTL$\star$
0.1	0.891	0.894	0.884	0.893	0.902	0.905	**0**.**907**
0.2	0.873	0.884	0.890	0.879	0.894	0.891	**0**.**896**
0.3	0.869	**0**.**885**	0.877	0.873	0.883	0.881	**0**.**885**

Bold number indicates the best performance, i.e., the lowest nMSE

#### Comparison of our algorithms

For comparing the efficiency of our two optimization algorithms, we set the maximum iteration number 20000, stopping criteria from $10^1$ to $10^{-7}$.We terminate the algorithm when the change of function value at two consecutive iterations is less than the stopping criteria. We compare the efficiency of our ADMM-based and AGM-based algorithms on the ADAS dataset. Refer to Fig. 2, clearly, the convergence rate of the AGM-based algorithm is much higher than the ADMM-based algorithm, this is consistent with the theoretical analysis. Both optimization algorithms have similar CPU times in the case of low accuracy, i.e., the stopping criteria $\in [10^1, \cdots , 10^{-3}]$, which may be related to the utilization of the inexact ADMM to improve the speed. However, in the case of high precision, the AGM-based algorithm is obviously much faster, which shows the efficiency of applying the decomposition property of the composite penalty in 6. We also find that the actual convergence speed of the ADMM-based algorithm is related to the choice of $\rho$. For example, the ADMM-based algorithm with $\rho = 2.5$, which is neither maximum (5) nor minimum (1), almost has the slowest convergence result. This property presents a challenge to design an algorithm based on ADMM for practical problems since we need to put some effort for selecting a $\rho$ with a proper value.

Then we study the classification problem on the Employee dataset by setting the maximum iteration number 1000. From another different point of view as the above part, we study the situation of the loss function value. Refer to Fig 3, the function value generated by the ADMM-based algorithm seems to be stuck at some random value and can not converge any more. The three specific ADMM-based algorithms with different $\rho$ converge to same value roughly, but with different convergence rates. Clearly, the AGM-based algorithm convergences better with a lower function value. More than that, as shown in Table 3, with the same number of iterations, the AGM-based algorithm has much less CPU time than the ADMM-based algorithms. To be specific, when we set the maximum iteration number 10, the AGM-based algorithm is $0.208/0.083 \approx 2.5$ times faster than the ADMM-based algorithm. And when the maximum iteration number is 100, the AGM-based algorithm is $26.5/6.92 \approx 3.8$ times faster than the ADMM-based algorithm.

We conclude the AGM-based algorithm is more efficient than the ADMM-algorithm for our approach, no matter in regression dataset or classification dataset. Note that in the following part, we choose the AGM-based algorithm to solve the ATMTL formulation 6.

#### Compare with baseline methods

To study the efficiency of our proposed ATMTL, we compete with the existing methods which consider the task relation structure, including task grouping methods: LTGO [23], VSTG [22]; the trace norm (low rank) methods: RMTL [7], MNorm [8] and the temporal method cFSGL [52]. We emphasize that we do not consider the models whose proximal operator has an analytical solution like TGL [51], which usually does not have good enough performance shown in Sect. 5.3. It is worth noting that these aforementioned methods do not have the same objective function and the concrete theoretical complexity is hard to compute. For example, LTGO and VSTG are both biconvex, which sets the challenge for a clear computational complexity. In order to compare the efficiency of each method as fairly as possible, we repeat the experiment 10 times with randomly selected parameters on a large-scale MMSE dataset and the average value is reported. We denote the AGM-based algorithm for solving our formulation as ATMLT and without using Lemma 4 as ATMTL-no.

**Fig. 4:**
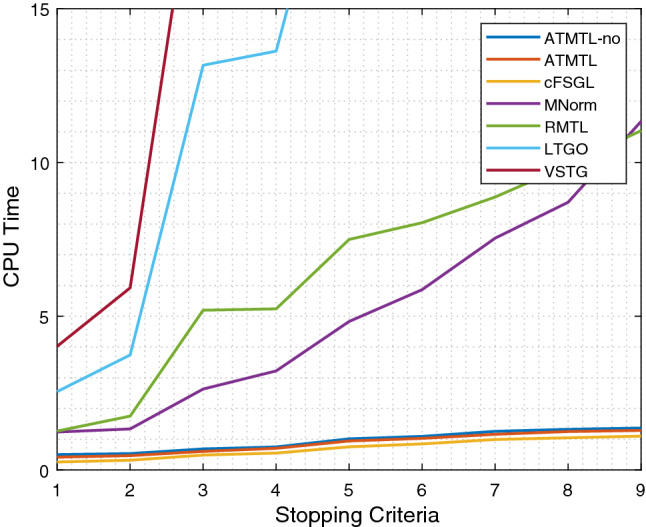
Comparison of the efficiency in terms of CPU time (s) on MMSE dataset. The stopping criterion is from $10^0$ to $10^{-4}$. When the change of the function value on two consecutive iterations is less than stopping criterion, we terminate the program

According to Fig. 4, we notice the two task grouping methods LTGO and VSTG do not have much efficiency may be due to the bi-convex objective function, especially the $l_{1,\infty }$ and k-support norms in VSTG which are both with additional computational effort. RMTL and MNorm, using trace norm with the complexity $\max (tp^2, t^2p)$ for computing the proximal operator, where *t* is the total number of tasks and *p* is the feature dimensionality. The third-order complexity needs additional computational effort and actually makes RMTL and MNorm not able to be scalable to large-size problems. ATMTL, ATMTL-no, and cFSGL have almost roughly the same efficiency since they are all based on the decomposition property [52]. Note that ATMTL is faster than ATMTL-no, demonstrating the effectiveness of our proposed Lemma 4, in which we directly compute the largest Lipschitz constant to avoid the computation of line search for choosing a proper step-size. We summarize that our ATMTL solved by the AGM-based algorithm has basically the highest efficiency among these methods.

### Effectiveness

#### Experimental setting

For evaluating the effectiveness of our approach, in this subsection, we terminate all the algorithms when the relative change of the two consecutive objective function values is less than $10^{-4}$. The reported experimental results are averaged over 10 random repetitions of the dataset. We separate the dataset with different ratios, and without special mention, the ratio is 0.8, which means we split the dataset into a training set and a test set of the ratio 8 : 2. We use the following normalized mean squared error (nMSE) [46, 54, 55] to evaluate the regression algorithms on the test set.

$$\begin{aligned} nMSE(Y, {\hat{Y}}) = \frac{\sum _{i=1}^{t}\Vert \varvec{y_i}-\hat{\varvec{y_i}}\Vert _2^2 /\sigma ^2(\varvec{y_i})}{\sum _{i=1}^{t}n_i}. \end{aligned}$$

For binary classification algorithms, the accuracy (ACC) is applied. All parameters are tuned via 5-fold cross-validation.

#### Temporal sparse group lasso

In order to show the effectiveness of our temporal sparse group Lasso (tsgL), we compare the several methods used for feature selection on Parkinson dataset. For a more comprehensive experimental analysis, we consider a different scenario with different task numbers. Competing methods include Lasso [37], group Lasso (gLasso) [44], $L_{1, \infty }$-norm (hLasso) [48], group Bridge (gBridge) [18], sparse group Lasso (sgLasso) [36] and Log-exp-sum (LES) [11]. Table 4 shows the details of the involved methods with the range of the hyperparameters.

According to Table 5, most of the methods like gLasso, spLasso, gBridge, LES that introduce both intra-group sparsity and inter-group sparsity have better performance than Lasso, since the latter does not consider the natural grouping of features.Note that hLasso perform badly, no matter how many task we have. Especially when the task number is 3, the nMSE achieves the highest value 1.265, corresponding to the poorest performance. It means Parkinson’s feature set does not have a clear hierarchical form. Our tsgLasso has the smallest nMSE (0.909, 0948, 0.948 with the task number is 2, 3, 4), i.e., the best performance, in all cases, which means the introduction of our AGTS is effective. We also notice that tsgLasso’s improvement, compared to sgLasso, continues to decrease as the number of tasks increases. The possible reason is the state of Parkinson’s patients is relatively stable, so our AGTS does not have extremely high improvement. Another possible reason is the influence of the trade-off of task relation created by our AGTS is getting weaker as the number of task goes up. We can solve this problem by setting different parameters. For example, the parameter $\alpha _i$ is set to represent the related degree between $(i+1)$-th task and all previous tasks. However, this method results in many hyperparameters for tuning which requires heavy computational cost and is not practical in the real world.

#### Global temporal smoothness

To evaluate our proposed global temporal smoothness assumption, we compare the performance of four penalties that focus on temporal smoothness assumption, including Laplacian-based penalty (Lbased) [51], fused Lasso based penalty (FLbased ) [52], our global Laplacian-based temporal smoothness penalty (GLbased), and global fused Lasso based temporal smoothness penalty (GFLbased), on MMSE dataset. Table 6 shows the details of the involved methods with the range of the hyperparameters.

As the experimental results shown in Table 7, we study the setting with different tasks number from 2 to 6, we notice both two penalties based on global temporal relation among multiple time points achieve clear improvement, demonstrating the effectiveness of the introduction of global temporal information. Especially when the number of tasks is 2, the nMSE arrives at the lowest value 0.678. Clearly, the Laplacian based smoothness methods Lbased and GLbased perform poorer than the fused Lasso based smoothness methods FLbased and GFLbased. It shows the effectiveness of the row decouple of model coefficient matrix *W*. It is worth noting that the same phenomenon as Table 5, the larger the task number is, the less improvement our novel global temporal smoothness penalties have.

#### Performance of ATMTL

For evaluating the performance of our novel ATMTL, we compare it with several baseline MTL methods whose details are in Table 8. Refer to Table 9, which shows the result conducted on COVID-19 dataset, we notice that both RMTL and MNorm using trace norm do not perform well, which is probably because using trace norm to introduce low-rank structure is not suitable for COVID-19 dataset.Also note the nMSE of LTGO and VSTG is average, which may be because there is no obvious task grouping in COVID-19 dataset. In addition, NC-CMTL and MTFLC have poor performance, maybe due to the focus on the noise level of tasks without taking into account the complex relation between tasks.TGL and cFSGL have lower nMSE than the above methods, the possible reason is they consider both feature selection and local temporal connections between tasks. cFSGL performs better than TGL, indicating the influence of intra-group sparsity.Note that our model basically has the lowest nMSE in the setting of task numbers equal 2 and 4, which indicates the global temporal relation has an important effect. Although cFSGL gets the best nMSE 0.713 when the task number equals 3, our ATMLT has a very similar result (nMSE = 0.714).

For the binary classification problem, we conduct the experiment on Employee dataset. Note that for classification problem, MTFLC and NC-CMTL are not suitable, so we discard them. The experimental results shown in Table 10 have some similarities with the results on COVID-19 dataset. Actually, we limit the sample number by setting different ratios. We emphasize under the scenarios with different ratios, our ATMTL has the best prediction accuracy in classification problems.

Both the results about the regression problem and classification problem demonstrate that our proposed ATMTL not only outperforms multiple baseline MTL models in terms of effectiveness; but also is basically the most efficient among methods that consider the task relation.

## Conclusion

In this paper, we proposed a novel MTL approach that simultaneously performs feature selection and adaptively captures the global temporal task relatedness. Our main assumption is for the class of progression problem, the state at the current time point is related to all previous time points. To be specific, we propose a temporal sparse group Lasso to allow simultaneous joint feature selection for all tasks and selection of a specific set of features for each task. And we present a global temporal smoothness to capture the complex temporal relatedness among multiple time points. Two algorithms, based on ADMM and AGM respectively, are designed. Experimental results on four real-world datasets demonstrate our approach not only outperforms existing baseline MTL methods in terms of effectiveness; but also is basically the most efficient among several methods which consider the task relation.

There are two interesting directions to improve the proposed approach in future work. First, considering the reduction of training time, we try to utilize the non-convex technique to reduce the number of hyperparameters of our approach. Second, introducing spatial information into our approach is expected to achieve higher capability.

## Data Availability

The datasets generated and/or analysed during the current study are available from the corresponding author on reasonable request.

## Footnotes

1 https://www.ibm.com/communities/analytics/watson-analytics-blog.
